# Intraoperative Ultrasound-Assisted Extent of Resection Assessment in Pediatric Neurosurgical Oncology

**DOI:** 10.3389/fonc.2021.660805

**Published:** 2021-04-21

**Authors:** Andrea Carai, Alessandro De Benedictis, Tommaso Calloni, Nicola Onorini, Giovanni Paternò, Franco Randi, Giovanna Stefania Colafati, Angela Mastronuzzi, Carlo Efisio Marras

**Affiliations:** ^1^ Neurosurgery Unit, Department of Neurological and Psychiatric Sciences, IRCCS Bambino Gesù Children’s Hospital, Rome, Italy; ^2^ School of Neurosurgery, University of Milan-Bicocca, Milan, Italy; ^3^ Neuro-Radiology Unit, Department of Imaging, IRCCS Bambino Gesù Children’s Hospital, Rome, Italy; ^4^ Department of Hematology/Oncology, Cell and Gene Therapy, IRCCS Bambino Gesù Children’s Hospital, Rome, Italy

**Keywords:** intraoperative ultrasound, neurosurgical oncology, extent of resection, brain tumor, children

## Abstract

Central nervous system tumors represent the most frequent solid malignancy in the pediatric population. Maximal safe surgical resection is a mainstay of treatment, with significant prognostic impact for the majority of histotypes. Intraoperative ultrasound (ioUS) is a widely available tool in neurosurgery to assist in intracerebral disease resection. Despite technical caveats, preliminary experiences suggest a satisfactory predictive ability, when compared to magnetic resonance imaging (MRI) studies. Most of the available evidence on ioUS applications in brain tumors derive from adult series, a scenario that might not be representative of the pediatric population. We present our preliminary experience comparing ioUS-assisted resection assessment to early post-operative MRI findings in 154 consecutive brain tumor resections at our pediatric neurosurgical unit. A high concordance was observed between ioUS and post-operative MRI. Overall ioUS demonstrated a positive predictive value of 98%, a negative predictive value of 92% in assessing the presence of tumor residue compared to postoperative MRI. Overall, sensibility and specificity were 86% and 99%, respectively. On a multivariate analysis, the only variable significantly associated to unexpected tumor residue on postoperative MRI was histology. Tumor location, patient positioning during surgery, age and initial tumor volume were not significantly associated with ioUS predictive ability. Our data suggest a very good predictive value of ioUS in brain tumor resective procedures in children. Low-grade glioma, high-grade glioma and craniopharyngioma might represent a setting deserving specific endeavours in order to improve intraoperative extent of resection assessment ability.

## Introduction

Several studies have demonstrated that extent of resection is a crucial prognostic factor for achieving the best outcome in neurosurgical oncology (1).

For this reason, previous investigations have focused on the possible contribution of intraoperative imaging techniques in improving surgical results (2). In this context, intraoperative ultrasonography (ioUS) is a promising tool to assist the surgeon in accomplishing several tasks, including target localization, volume and margin delineation, real-time brain shift evaluation and assessment of extent of resection (3, 4). Progressive technological improvement has allowed the differentiation of distinct tissue patterns, including necrosis, hemorrhage, and cystic components of tumors (5–7). Moreover, ioUS offers significant advantages in terms of availability, versatility and costs in comparison to other intraoperative imaging modalities, such as MRI and CT (8).

However, available evidence is mainly based on adult case series, which might fail to account for population specific features of pediatric disease (9, 10).

In this study, we report our experience on the use of ioUS in series of pediatric patients undergoing brain tumor resection.

## Materials And Methods

All patients undergoing ultrasound assisted brain tumor resection at the Neurosurgery Unit of the Bambino Gesù Children’s Hospital from January 2018 to June 2020 were included in the study.

Extent of resection was evaluated according to latest recommendation of the International Society of Pediatric Oncology (SIOP), integrating a surgical grading with a MRI grading. Surgical impression on resection was therefore graded from SR0 to SR3 as follows: SR0 (complete resection), SR1 (rim-like residual), SR2 (bulky residual), SR3 (biopsy). Radiological assessment was graded MR0 to MR3: MR0 (complete resection), MR1 (rim-like residual ≤ 3 mm), MR2 (residual > 3 mm in any section), MR3 (residual > 50% of initial volume) (11) **(** **Table 1** **)**

**Table 1:** Extent of resection as evaluated intraoperatively and on postoperative contrast-enhanced MRI to be performed within 48 h (max, 72 h) after surgery.

SR 0	Total resection, no residue
SR 1	Suspected residue, possible local invasion
SR 2	Solid residuum (to be defined by postoperative MRI)
SR 3	Tumor volume unchanged, biopsy
MR 0	No visible tumor
MR 1	Rim enhancement or signal abnormality (matching the tumor) at the operation site only (“Rim”), ≤ 3 mm in any of the dimensions and equivocal for tumor residue
MR 2	Residual tumor measuring > 3 mm in all 3 dimensions (greater than MR1, less than MR3)
MR 3	No significant change to preoperative tumor size (“minimal change”)

Adapted from Gnekow (11).

Planned resection (pSR) was defined during multidisciplinary neuro-oncology board meetings. Unless a bioptic procedure was indicated, maximal safe resection was always planned.

**Figure 1:**
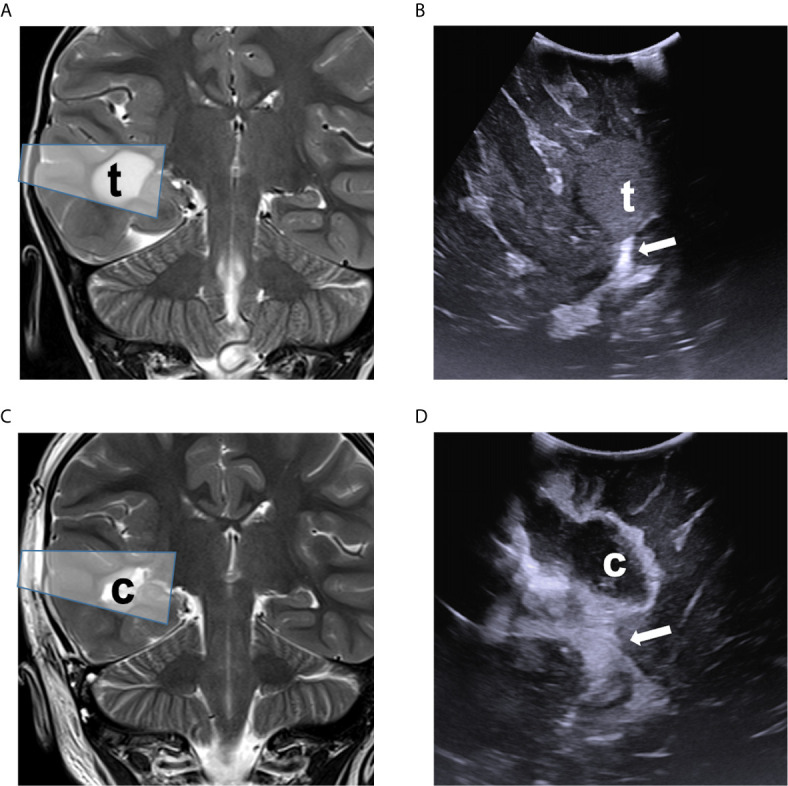
Comparison of ioUS with pre- and post-operative MRI images of a right temporal low-grade glioma. Pre-operative **(A)** and post-operative **(C)** coronal T2-weighted MRI sequence demonstrating the lesion (t) and surgical cavity (c) with ioUS approximate field of view (shaded box). Intraoperative US view of the same is shown before dural opening **(B)** and after resection **(D)**, documenting the spatial relationship with the choroid plexus (white arrow).

**Figure 2:**
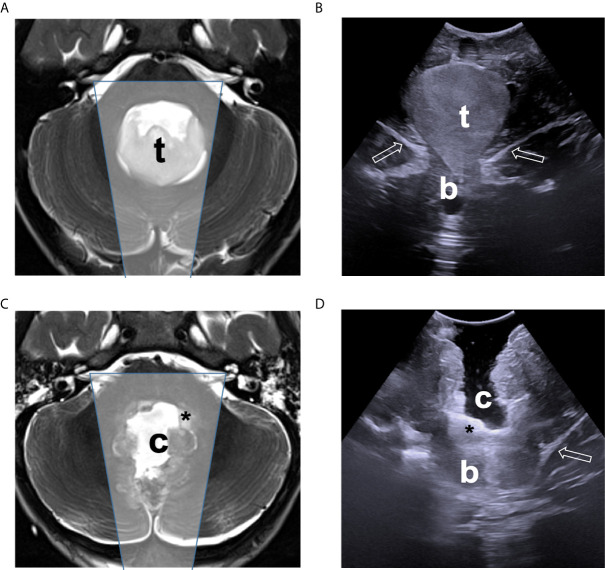
Comparison of ioUS with pre- and post-operative MRI images of a IV ventricle low-grade glioma. Pre-operative **(A)** and post-operative **(C)** axial T2-weighted MRI sequence demonstrating the lesion (t), and the surgical cavity (c), tumor residue (asterisk) respectively, with approximate ioUS field of view (shaded box). Intraoperative US view of the same is shown before dural opening **(B)** and after resection **(D)**, documenting the presence of a small tumor remnant (asterisk) which was intentionally left in place to avoid damage to the IV ventricle floor structures.

All children underwent navigated craniotomy (Medtronic S7) and microsurgical resection of the lesion. Intraoperative ultrasound (BK 5000, BK Medical, Peabody, MA) equipped with a 5- to 10-MHz convex probe (Craniotomy probe N13C5, BK Medical) was used before and after dural opening to confirm the relationship of the lesion to brain landmarks (**Figures 1B** and **2B**), during resection at surgeon’s discretion, at the end of resection to confirm the microsurgical impression of reaching the planned resection (SR) (**Figures 1D** and **2D**). To reduce inter-operator variability, intraoperative evaluations in our series were only performed by three surgeons sharing the case series, each having at least a 5-year experience in ioUS use (AC, ADB, CEM).

Post-operative imaging was performed on a 3T Siemens MRI machine (**Figures 1A, C**, **2A, C**). All scans were reviewed by an experienced pediatric neuroradiologist (GSC) blinded to the intraoperative impression.

The agreement between intraoperative ultrasound evaluation and MRI was measured with Fleiss’ kappa agreement (12). The Chi-square test was used to analyze associations between categorical variables, which were expressed as absolute numbers and percentages. Multivariate logistic regression analysis was performed to identify predictors for discordance between intraoperative ultrasound evaluation and MRI (the model included as variables age at intervention, diameter of the lesion, localization of the tumor and surgical position). Statistical analyses were performed using GraphPad Prism, version 9.0 (GraphPad Software, San Diego, California, USA, www.graphpad.com).

IRB approval was obtained for this retrospective study, including waiver of consent from participating patients.

## Results

Our series (**Table 2** **)** included 154 patients, mean age was 8.6 years with a median of 8.2. Average tumor diameter was 36.18 mm with a median of 34 mm.

**Table 2:** Study population.

Features of patient population and disease			
Average Age	Years:	8, 6	
Sex	M	93	60,39%
	F	61	39,61%
Average Diameter	mm	36, 18	
Histology	LGG	81	52,60%
	HGG	10	6,49%
	Embryonal	22	14,29%
	Ependymoma	12	7,79%
	Craniopharyngioma	6	3,90%
	Choroid P. tumors	3	1,95%
	Other	20	12,99%
Site	PCF	66	42,86%
	Hemispheric	58	37,66%
	Intraventricular	12	7,79%
	Pineal	8	5,19%
	Sella	6	3,90%
	Thalamus	4	2,60%
Patient position during surgery	Prone	84	54,55%
	Supine	49	31,82%
	Sitting	21	13,64%

The most frequent tumor location was found to be the posterior fossa (46 cerebellum, 6 IV^th^ ventricle, 10 brainstem, and 4 cerebello-pontine angle). Hemispheric lesions were 58: 19 frontal, 29 temporal, 7 parietal, 2 cingular and 1 occipital. Additional deep tumor locations were less frequent and included: 12 intraventricular, 4 thalamic, 6 sellar and 8 pineal.

Patient positioning during surgery was determined based on lesion location, therefore the most frequently used was the supine position (84). In posterior fossa and pineal region tumors, the prone (49) and sitting (21) position were also used.

Most frequent histology was low-grade glioma (81), followed by medulloblastoma (17), ependymoma (12) and high-grade glioma (10). Additional tumor subtypes included craniopharyngioma (6), germ cell tumors (5), choroid plexus tumors (3) and other less common tumors (20). Due to heterogeneity of the histologies and tumor locations, for statistical purposes, we had to group the cases into broader categories (**Table 2**)

In children with a central nervous system tumor, the most frequently planned procedure was a complete resection (pSR0 in 111 cases) and a “near total” resection (pSR1 in 15 cases). In 26 cases a debulking procedure was planned (pSR2), while a biopsy was rarely indicated (pSR3 in two cases).

At the time of surgery, extent of resection was estimated by integrating microscopic evidence, neuronavigation information and intraoperative ultrasound assessment. Ultrasound assessment was possible in all cases, despite some technical limitations were anticipated in selected settings including parietal lesions (10) sitting position (13) and large tumor size (14).

Intraoperative assessment confirmed achievement of a planned SR0 in 95% of cases (SR0 106/111, SR1 5/111), pSR1 in 100% (15/15), pSR2 in 96% (SR2 25/26, SR3 1/26) and pSR3 in 100% of cases (2/2). In five cases the surgery was stopped despite the fact the assessment of a lower than planned EOR (SR1 instead of pSR0). Four of these patients had a LGG arising from or infiltrating the brainstem, the other patient had a recurrent posterior fossa ependymoma with infiltration of the lower cranial nerves which was not fully predictable on preoperative imaging. The child in which a biopsy was obtained instead of a subtotal resection (SR3 instead of pSR2) had a very large (133 mm in diameter) high-grade glioma infiltrating the third ventricle walls and thalamus bilaterally.

Post-operative MR confirmed intraoperative assessment in 87% of cases, stratified as follows: SR0 in 92% of cases (MR0 97/106, MR1 5/106, MR2 4/106), SR1 in 47% of cases (MR0 in 1/19, MR1 in 9/19, MR2 in 9/19), SR2 in 100% (25/25) and SR3 in 100% (MR3 3/3).

Overall, in this cohort of pediatric brain tumors, when used to assess the Extent of Resection as compared to early post-operative MRI, ioUS was found to have sensibility of 86%, specificity of 99%, negative predictive value of 92% and positive predictive value of 98%.

Concordance analysis between intraoperative ultrasound evaluation and MRI showed substantial agreement (kappa = 0.758) (12). In details, intraoperative ultrasound evaluation underestimated tumor residual. Overestimation occurred only in a single case. Low-grade gliomas (underestimation: 15/81, 18.5%), high-grade gliomas (underestimation: 2/10, 20%) and CRF (underestimation: 1/6, 16.6%) were associated with US underestimation (p = 0.034). In order to correct for confounding factors, multivariate logistic regression analysis was performed, showing only histology as associated with discordance of the two imaging tests (odds ratio, 1.604; 95% CI, 1.126–2.623; p = 0.0234).

Bivariate statistical analysis did show a statistically significant correlation between ioUS failure to accurately assess residual tumor and histology. No correlation was found for other clinical variables, including age, tumor diameter, lesion location, and patient positioning during surgery (**Table 3**).

**Table 3:** Multivariate statistical analysis based on patients’ age, tumor diameter, histology, site, and position during surgery.

	p value
Age	0.7505
Diameter	0.8741
Histology	**0.0234**
Site	0.9966
Position	0.3713

Bolding is meant to underline the only statistically significant value.

## Discussion

Intraoperative imaging is an emerging tool in the neurosurgical armamentarium with a growing body of evidence to support its advantages for lesion targeting and extent of resection evaluation.

In the setting of pediatric neurosurgical oncology, control over the extent of resection is paramount. In this scenario, the introduction of real time intraoperative imaging, in addition to direct inspection of the microsurgical field integrated with neuronavigation data and intraoperative monitoring information has the potential to significantly improve surgical orientation. Indeed, while the aim of surgery is generally to achieve complete resection, in particular cases this may not desirable, making precise intraoperative assessment of residual disease a fundamental tool to tailor surgical resection.

The neuro-oncological pediatric population has several peculiarities when compared to adults, including a larger variety of histological subtypes and frequent lesion location in the posterior fossa. Therefore, generalization of available evidence concerning the use of ioUS, mostly derived from the adult population, might not be obvious.

As mentioned above, when used to assess the Extent of Resection compared to early post-operative MRI, in this series ioUS was found to have sensibility of 86% negative predictive value of 92%, with a specificity of 99% and positive predictive value of 98%. This trend for higher PPV than NPV has also been found in adults (15), underlying a residue found at ioUS is more likely to result in MRI evidence of tumor residue than negative ioUS is to result in radiologic GTR.

The data concerning the use of ioUS as an aid in detecting tumor residues in pediatric brain tumor resection is sparse and based on small series. Even more scattered is evidence regarding clinical variables associated to ioUS diagnostic yield, including lesion site and histology.

Smith and colleagues discussed the use of ioUS in resection of pediatric brain tumors: in a series of 62 patients, GTR was planned in 82%. Surgery was stopped when microscopy and ioUS demonstrated complete resection. In 71% of the patients, the GTR was subsequently confirmed by postoperative MRI, while in 11% a residue was diagnosed with MRI which ioUS failed to detect. Notably, the specificity of iOUS appeared to be particularly low in parietal tumors (55%), which the authors did not offer possible explanations for (10).

In a mixed cohort of children and adults, the same group described 42 false ioUS-based diagnoses of GTR out of 217 intended GTR procedures (19.35%). False negatives occurred mostly in the setting of surgery for glioblastoma possibly as a result of the difficulties in detecting the margins of these highly invasive lesions. No information concerning the location of the false negatives was provided, nor stratification of the results based on age (2).

El Betagy and colleagues published two papers concerning ioUS use in brain tumor resection in children. In the first one they described 25 patients, 14 of which underwent GTR with no additional data about planned extent of resection. The ability of ioUS in detecting tumor residue was claimed to be comparable to that of MRI (16). In a follow-up paper, 60 patients operated for posterior cranial fossa lesions in the prone position were divided into two groups, 30 to be operated with the aid of ioUS and 30 without. The use of iOUS resulted in a 16% increase in GTR achievement (96% vs 80%), while allowing a lower incidence of cerebellar mutism (3% vs 20%) without significant increase of the operative time. They reported ioUS usefulness in detecting residue in the region of the rostral vermis and the lateral recesses of the fourth ventricle (17). No information was provided concerning the patients’ randomization process.

Concerning pediatric posterior fossa lesions, a paper by Nagaty and colleagues described 23 surgeries performed with the aid of ioUS, in 11 of which GTR was achieved. The accuracy of ioUS was not compared to postoperative MRI, beside the fact that the average size of residuals diagnosed by ioUS and by MRI were similar (18).

Ulrich and colleagues described a series of 22 patients, in 19 of which a GTR was planned. On postoperative MRI, out of the 19 planned GTR procedures, a residual was diagnosed in a single case of IV ventricle medulloblastoma, which the iOUS failed to detect.

Our data confirm the high sensibility and specificity of ioUS in detecting the extent of residual disease in a pediatric brain tumor series in what is, to the best of our knowledge, the largest pediatric series published to date.

The pivotal role of accurate assessment of residual tumor is in children is underlined by the introduction by the International Society for Paediatric Oncology (11) of a new scale to quantify both the operator’s assessment and the post-operative imaging data. Our decision to assess residual disease according to this classification might account for some of the differences in extent of resection rate compared to previously reported series.

In our series, ioUS underestimated resection in 18 cases (12%), Notably, in five (3%) cases, it suggested a complete resection (SR0) had been accomplished while MR later showed a linear residual smaller than 3 mm in diameter (MR1) and in nine (6%) cases, it suggested a linear residual (SR1) instead of a nodular one (SR2). Further, 4 (3%) cases ioUS suggested complete resection (SRO) while a nodular residual (SR2) had been left behind. Notably, even if all patients were operated with the aid of intraoperative monitoring, resection was never interrupted because of neurophysiological data.

We did not find significant association between residual underestimation and either prone, supine or sitting positioning, While the inability to fill the tumor cavity with saline in sitting positioning has raised questions about ioUS reliability in this setting (13), the sitting position does not appear to correlate with false negatives in our experience.

A well-described technical pitfall of ioUS in the neuro-oncological setting is presence of artifacts when exploring tissue surrounding large cavities, due to the difference in sound propagation between saline solution and brain (14). Possible countermeasures include the use of small probes inserted in the surgical cavity, at the price of a limited field of view (14) and ongoing development of acoustic coupling gels as saline alternatives (19). Despite these concerns, in our analysis, tumor size did not correlate with ioUS failure to detect lesion residue.

Anecdotally, the single case of residue overestimation (0.6%) in our series was a large frontal tumor in which artifacts from tissue manipulation where misinterpreted as linear residual disease (SR1) not confirmed at the post-operative MR (MR0).

The only variable significantly associated with an unexpected tumor residue on the postoperative MRI was histology (p = 0.0234). In particular, all the false negatives in this series were LGG, HGG, and craniopharyngioma (CFR).

Most of the literature concerning ioUS as an aid in residue identification, which stems from mostly adult series, compares ioUS sensitivity in is generally reported to have higher sensitivity in the detection of residues of HGG than LGG (15, 20). In this series, while certain histologies were associated with residue, no significant difference was apparent between LGG and HGG. We believe similarity in echogenicity and microscopic appearance to brain parenchyma in the case of the former and peritumoral edema and infiltrating pattern, typically found in the latter, might have lessened the ability to distinguish the tumor remnants from the surrounding tissue. Identification of tumor residues in the sellar and parasellar region carries unique challenges due to the geometry of the cavity with respect to the major tumor axes and the high rate of artifacts due to the closeness of osteo-dural and vascular structures, while we did not find a statistical significance for tumor residue in the sellar region compared to other tumor locations, we believe these factor did play a role in the single case of residue in a CFR.

The low sensitivity of ioUS in parietal lesions reported by Smith and colleagues was not apparent in this series (10). Tumor location indeed did not associate with undetected residue on multivariate analysis.

While lesion location did not reach significance on multivariate analysis, the subgroup in which ioUS failed to accurately assess residual showed a high percentage of brainstem infiltrating lesions (50%) and posterior temporal lobe location with tumor residual on the wall of the resection cavity (49%). Intriguingly, these two conditions found in 89% of cases of underestimation of tumor residue share some technical challenges for ioUS despite US probe positioning at the top of the resection cavity. We speculate that inaccuracy in the posterior fossa might depend on peculiar echogenicity of brainstem and proximity of bone walls, while residual location on a wall of the resection cavity, artifacts from ventricular structures, proximity of middle fossa floor and tangential direction of the US probe to the surgical cavity might contribute in temporal lobe resections.

We speculate that the use of more advanced US techniques, such as contrast-enhanced (21) and navigated (22) ultrasound, which were not used in this series, might allow an even more reliable assessment of the extent of resection, as a growing body of evidence suggests.

## Conclusions

Maximal safe resection of brain tumors is a critical step of treatment in the pediatric population. Intraoperative extent of resection can be accurately assessed by ioUS in the vast majority of cases.

Further technical refinement and application of additional intraoperative advanced visualization tools might help overcome this limitation contributing to a more precise intraoperative residual detection in the future.

## Data Availability Statement

The original contributions presented in the study are included in the article, further inquiries can be directed to the corresponding authors.

## Ethics Statement

The studies involving human participants were reviewed and approved by the Institutional Review Board at the Ospedale Pediatric Bambino Gesù. Written informed consent from the participants’ legal guardian/next of kin was not required to participate in this study in accordance with the national legislation and the institutional requirements.

## Author Contributions

CM: manuscript conception and revision. AC and AD: data revision and interpretation, manuscript drafting, and revision. TC: data collection and revision and manuscript drafting. GP and NO: data collection. GC: imaging data interpretation. AM and FR: manuscript revision. All authors contributed to the article and approved the submitted version.

## Funding

This research was funded by the Ministero della Salute- Ricerca Corrente to Ospedale Pediatrico Bambino Gesù, IRCCS.

## Conflict of Interest

The authors declare that the research was conducted in the absence of any commercial or financial relationships that could be construed as a potential conflict of interest.
